# Supplementary material for: Are carbon emissions trading and green financial instruments synergistic? -Comprehensive quantitative research based on content analysis
Source: PLoS One. 2024 Mar 7;19(3):e0298601. doi: 10.1371/journal.pone.0298601 (PMC10919601; doi:10.1371/journal.pone.0298601)
Supplement: S1 Data — (ZIP) [file pone.0298601.s001.zip › Data/地级市Z_F工作报告.zip!/╜¡╬≈/╙Ñ╠╢╩╨/└┤╫╘╣1⁄2╓┌║┼í╛┬φ┐╦╩2╛▌═°í┐_2005.docx]

各位代表：

现在，我代表市人民政府向大会作政府工作报告，请予审议，并请市政协各位委员和列席会议的同志提出意见。

一、2004年政府工作的简要回顾

2004年，在省委、省政府和市委的正确领导下，全市人民以邓小平理论和“三个代表”重要思想为指导，深入贯彻党的十六大和十六届三中、四中全会精神，坚持科学发展观，同心同德，善谋实干，排难而进，全面和超额完成了市六届人大一次会议确定的各项任务，经济建设迈上了新台阶，社会事业取得了新成就。

（一）认真落实宏观调控政策，国民经济保持快速健康发展

过去的一年，我们坚决贯彻国家宏观调控政策，控制钢铁和水泥等部分行业过度投资，认真清理固定资产投资项目，治理整顿开发区和土地市场，继续鼓励符合国家政策的产业做优做强，努力克服制约经济增长的不利因素，国民经济在宏观调控中健康发展，在整顿规范中稳步提高。全市完成生产总值96.37亿元，比上年增长16.8%，人均生产总值首次超过1000美元，其中：一产增长7.5%,二产增长22.7%，三产增长14.3％，三次产业的比重为16.2：49.2：34.6。财政总收入10.66亿元，增长23.5%，人均财政总收入达到1000元；其中地方财政收入5.29亿元，增长18.1%。全社会固定资产投资43.92亿元，增长42.1%，增幅比上年回落13.2个百分点。城镇居民人均可支配收入达7723元，增长10.1%，农民人均纯收入3265元，增长20.2%。社会消费品零售总额28.59亿元，增长15.0%。上述主要指标的增幅均高于全省平均水平，排位明显前移。

惠农政策全面落实，农业丰收农民增收。组织千名干部进村入户，宣讲中央和省委一号文件，调动了广大农民种粮的积极性,进一步形成了支持农业的合力。严格兑现各项优惠政策，全市调减农业税及其附加1759万元，农业税灾欠减免470万元，发放粮食直补和良种补贴资金2488万元。继续加大农业投入，安排农业基础设施建设资金4155万元，农业综合开发资金1503万元，新增园田化面积2500亩，除险加固病险水库42座，完成人工造林9万亩。加大了农业产业化龙头企业的扶持力度，辐射带动作用进一步增强。取得了抗击禽流感的胜利，战胜了持续严重干旱。由于政策好、粮价高、人努力、天帮忙，去年农业总产值完成27亿元，增长10.4%，粮食播种面积达到156.74万亩，增长12.9%，粮食总产量达到11.24亿斤，增长17.7%，粮食总产和农民人均增收创历史最好水平。

坚持扶优扶强，工业实现三年翻番。驻鹰大中型企业产能效益稳步提高，江西铜业集团公司销售收入超过150亿元，继续成为全省工业企业的排头兵。市属骨干企业以市场为导向，以科技为依托，发展的内生动力不断增强，经济效益明显提高。工业园区初具规模，成为新的经济增长点。全市工业园区累计完成基础设施投资6.6亿元，园区落户企业增加到153家，其中投资一千万元以上企业69家。耐乐铜业、安固铜材、金泉铜业、亚菲达铜业、中轻刀剪、江西铜材、星耀铜业等一批企业竣工投产。铜材加工、精密机械、医药、化工、眼镜等特色产业集群正在形成。积极推进科技兴企战略，产品品牌进一步做大。贵冶牌阴极铜获“中国名牌产品”称号，“施大壮”成为全国磷复肥行业首个驰名商标，三川牌水表获国家免检产品殊荣。全年完成更新改造投资13.56亿元。全市实现工业企业销售收入201亿元，增长45.6%，工业增加值43.57亿元，增长22.9%，实现利税19.89亿元，增长120%，均在2001年基础上实现了三年翻番。

旅游经济不断升温，大旅游格局开始形成。为了打造旅游精品，正式启动了创建“中国优秀旅游城市”工作。不断更新促销手段，举办了北京龙虎山旅游推介会，开行了旅游专列，旅游大蓬车驶入江浙，旅游宣传形式多样。团队游客增长较快，占游客总数上升到46%。鹰潭市首届旅游商品暨第二届优质农产品博览会取得成功。开通了江铜工业游、贵溪冷水耳口绿色生态游、余江韬奋故里游、白鹤湖农业生态游等一批新的旅游线路。九曲洲农业观光园被评为全国农业旅游示范点。旅行社和星级宾馆规模扩大，档次提高，旅游接待能力增强。龙虎山景区全年接待游客121.2万人次，增长58%，直接收入3850万元，增长48%。

商贸流通日益繁荣，财税金融运行平稳。专业市场经营趋旺，商品流通辐射范围扩大。房地产需求旺盛，全年商品房竣工面积46.43万平方米，城市居民人均住房面积有所增加。通讯、邮政、保险、餐饮和中介服务等第三产业有了新的发展。加大了多渠道筹资力度，争取国债建设资金1.39亿元。省工商银行授信的1.8亿元贷款额度已全部到位。税务部门努力培植税源，坚持依法征税，切实做好服务。财政部门精心理财，在确保法定增长的前提下，逐步消化历年旧欠，集中财力确保重点支出，做到了收支平衡。居民消费价格指数为103.3%。金融机构年末存款余额102亿元，增长12.3%，贷款余额110亿元，增长11.1%，均首次超过100亿元。

（二）改革开放不断深入，经济外向度逐步提高

招商引资有了新的突破。实际引进外资3578万美元，增长33.6%；实际引进内资47.0亿元，增长49.1%。引进外资总量首次在全省设区市排位前移。投资1000万元以上的项目有144个，增长16.1%。工业项目实际进资额占总额的63.5%。成功地举办了厦门招商会、闽粤赣十三市党政领导第九次联席会、闽浙赣皖九方经济区二届九次联席会和首次民间招商联谊会。外贸、外经工作取得新成绩，外贸出口2431万美元，增长17%。对外交往进一步扩大，贵溪市与日本和歌山县清水町签订了友好城市合作意向。

各项改革继续稳步推进。农村税费改革不断深化，农民人均负担由税改前的83.96元减少到28.51元。粮食流通体制改革深入推进，粮食购销市场全面放开，市场主体多元化格局基本形成。采取积极措施鼓励、支持和引导非公有制经济加快发展，全年新增个体工商户、私营企业分别为3297户和264户，其中注册资本1000万元以上的达56户。个私经济提供的生产总值、税收和就业岗位分别占全市的8.59%、17.57%和14.76%。整顿和规范市场经济秩序工作力度加大，食品、药品、烟草、建材、农资等专项整顿成效明显。深入推进行政事业单位改革。顺利完成了铁路学校、幼儿园和市新闻出版局移交地方管理工作。贵溪市农村新型合作医疗制度改革试点初见成效。农村信用联社改革进展顺利。

（三）基础设施建设全面提速，城市面貌实现大变样

全力以赴抓好重点工程建设，交通、电力、水利和社会事业等薄弱环节得到加强。举全市之力支持国家和省重点工程建设，贵溪电厂2×30万千瓦机组扩建、浙赣铁路电气化改造、黄金埠电厂铁路专用线建设等工程进展顺利。龙虎山龚资线、余江邓山线建成通车，全年修建各类农村公路304.6公里，54%的行政村修通了水泥路。贵溪罗河110千伏变电站建成投产。继续实施了余江中潢圩堤、团湖圩堤等圩堤的除险加固，开工建设了白塔渠挖潜配套改造工程，完成了贵溪白鹤湖农业生态观光园一期工程建设，市体育中心、市人民医院门诊医技大楼和市消防指挥中心开工建设。市、县两级疾病预防控制中心完工或即将完工。鹰潭新汽车站建成并投入使用。贵溪市、余江县出租车全部换型更新。其它各项基础设施和社会事业建设项目也取得重大进展。

围绕落实市委提出的城市建设三年实现大变样的目标要求，城市建设有了标志性变化。东湖景观一期工程、鹰东大道建设、沿江大道C段建设、梅园大道及军民路综合改造工程、平安路改造、解放路北段贯通工程全部竣工并投入使用。51号路、院里路即将建成。污水处理工程、垃圾填埋场、江南水厂扩建和管道燃气工程进展顺利。小街小巷硬化亮化完成投资250万元，进一步改善了社区居民的居住环境。基础设施的完善，进一步拓展了城市的发展空间，高桥新区、梅园新区成为投资建设的热点，城市建成区面积扩大到20.6平方公里。区域中心城市的地位进一步巩固。贵溪320国道改造、城南滨水休闲文化广场建设和余江果喜大道改造等工程竣工，有力地提升了城区品位。城市规划和管理水平不断提高，实行了城市规划公示制，扩大了市民参与度。开通了城建“12319”热线，进一步完善了便民服务。城市执法进一步规范，市容环境更为整洁。

（四）各项社会事业协调发展，精神文明建设全面推进

科技工作取得新成果。我市被科技部批准为国家新材料产业化示范基地和全国首批科技进步示范市。获国家级科技进步二等奖1项，省科技进步奖4项。教育投入不断加大。筹措校舍建设资金2173万元，改造中小学校危房4.95万平方米，危房比例由年初的8%下降至年底的6%。优质高中建设稳步推进。职业教育发展加快。教育教学质量不断提高。圆满完成了全省高招录取服务保障工作。进一步做好土地保护和利用工作。严格执行国家土地政策，经营性用地实行了招标、拍卖和挂牌有偿公开出让。环境保护力度加大，查处了一批超标排污企业，关停了一批污染严重企业。计划生育基层基础工作进一步巩固，整治性别比例失调专项工作力度加大，人口自然增长率为8.03‰，计划生育率为93.3%，均控制在省下达目标之内。卫生工作不断加强，稳步推进城镇医药卫生改革，建立健全了四级公共卫生防疫信息网络。经济普查各项工作有序开展。精心创作了一批文艺节目，举办了首届艺术节，社区群众性文艺活动丰富多彩。余江、月湖所有乡镇都架通了有线电视光缆。成功地举办了市第五届运动会，推动了竞技体育和全民健身运动的蓬勃发展。开展了争创全国双拥模范城活动，进一步落实了拥军优属政策和优惠措施，拥军优属拥政爱民活动成效显著。精神文明创建活动有新起色。“告别陋习、树立新风”活动深入开展，市民素质进一步提高。涌现出全国精神文明建设先进单位20个，省级文明单位40个。一批先进单位和先进个人受到表彰。审计、人事编制、档案、人防、民族宗教、外事侨务、气象服务、防震减灾、民兵预备役、老龄、残疾人等各项事业健康发展。

（五）做好就业和社会保障工作，切实解决群众困难

就业和再就业工作力度继续加大。建立了就业、再就业帮扶挂点制度。举办了春、夏、秋三季招聘洽谈会。全市城镇新增从业人员1.94万人，实现下岗失业人员再就业0.53万人，对外输出劳务人员15.85万人，城镇登记失业率为3.6%。社会保障制度不断完善。认真做好三条保障线衔接工作。全面超额完成了省政府下达的扩大就业和“两个确保”目标任务。建立城市低保配套帮扶机制，落实了低保特困户医疗、住房和水电方面的优惠政策。及时将农垦、森工企业特困群众纳入了城市低保。全市2.96万人享受了城市最低生活保障，基本做到了应保尽保。养老保险、失业保险和医疗保险的覆盖面进一步扩大。扶贫济困工作广泛开展。全市有2.39万人享受了农村特困群众社会救助，发放救助资金234.8万元，下拨救灾款152万元。开展了“慈善一日捐”和“扶贫济困送温暖”等多种形式的社会捐赠活动。全面实施五保供养“敬老工程”和第三批“星光计划”建设项目，新建改造敬老院10所1.1万平方米。高度重视和维护人民群众切身利益。积极开展农民工工资清欠工作，切实维护被征地农民和城市拆迁居民的合法权益，农村征地补偿费全部付清。

（六）坚持依法治市，全力维护社会稳定

认真学习贯彻《行政许可法》，行政审批制度改革取得成效。市本级清理行政许可项目479项，取消79项；清理行政实施主体49个，取消不具备实施主体资格4个。行政许可收费管理进一步规范，行业不正之风得到有效遏制。行政监察、监督和查处力度加大，经济发展环境明显改善。工程招投标、企业产权交易、经营性土地公开出让、政府采购等工作更加规范。市联合办证服务中心服务水平有了新的提高。坚持政务公开，重大决策广泛听取人民群众意见，提高了民主、科学决策水平。开展了“法律进村入户”、“法律进社区”活动，干部群众法律意识和依法办事能力得到提高。人民群众参政议政意识进一步加强。办理人大建议35件，政协提案173件，均全部办结。处理信访稳定问题力度不断加大，许多不稳定因素解决在萌芽状态。社会治安综合治理成效明显。城市防控体系建设更趋完善，科技强警力度进一步加大，农村派出所条件大为改善。完成了市看守所供水工程的改造。开展了“大练兵”活动，公安队伍整体素质稳步提高。全市刑事案件发案率下降，破案率提高。安全生产形势总体平稳，食品放心工程成效明显，人民群众的安全感进一步增强。

各位代表，过去的一年，是我市改革开放和经济建设取得重大进展的一年，也是我市力求加速崛起、实现小市有大作为取得阶段性成就的一年。这些成绩的取得，是全市人民在市委的正确领导下，认真贯彻党中央、国务院和省委、省政府的大政方针，抢抓机遇、团结拼搏、扎实工作的结果；是市人大依法监督、市政协民主监督和积极参与的结果；是各民主党派、工商联、群众团体和驻鹰单位、人民解放军、武警官兵以及离退休老同志共同努力和大力支持的结果。在此，我代表市人民政府，向全市人民，向所有关心、支持我市经济建设和社会发展的同志们，表示衷心感谢并致以崇高敬意！

在肯定成绩的同时，也要清醒地看到，我市经济总量仍然偏小，经济结构调整任务繁重；农民增收基础比较薄弱，地方工业发展亟待加快；就业还不够充分，部分低收入群众生活比较困难；少数历史遗留问题尚未完全解决，影响社会稳定的因素依然存在；政府职能转变尚未完全到位，执政能力有待进一步提高。这些问题必须引起我们的高度重视，采取切实有效的措施，认真加以解决。

二、2005年政府工作的总体要求和预期目标

2005年是全面实现“十五”计划、衔接“十一五”发展的关键一年。今年我市经济社会发展的总体要求是：以邓小平理论和“三个代表”重要思想为指导，认真贯彻党的十六大、十六届三中、四中全会和中央经济工作会议以及省委、市委全体（扩大）会议精神，坚持以科学的发展观统领经济社会发展全局，着力推进改革开放，切实转变经济增长方式，进一步巩固农业基础地位，继续抓好工业、城市建设和旅游三项重点工作，促进经济和社会事业持续协调快速发展，全面推动“小康鹰潭”、“和谐平安鹰潭”的构建进程，为实现全市综合经济实力再上新台阶奠定坚实的基础。

主要预期目标是：全市生产总值108.9亿元，增长13.0%；财政总收入12.11亿元，增长13.6%，其中地方财政收入6.0亿元，增长13.4%；全社会固定资产投资53.6亿元，增长22.0%；社会消费品零售总额增长12.5%；居民消费价格总指数涨幅控制在4%以内；城镇居民人均可支配收入增长7.0%；农民人均纯收入增长6.1%；城镇登记失业率控制在4.2%以内；人口自然增长率控制在省下达的目标以内。

实现上述目标，政府工作在指导思想上要突出“四个坚持不懈”：坚持不懈地落实科学发展观。我市近几年逐步完善了符合鹰潭实际的发展思路，创造了一个比较好的发展环境，要保护好、引导好、发挥好各方面加快发展的积极性。牢牢把握发展机遇期，注重科学发展，协调发展，统筹兼顾，促进全市经济社会更快更好地可持续发展。坚持不懈地促进经济增长方式的转变。从战略高度落实中央“区别对待，有保有压”的宏观调控政策和“双稳健”的财政、货币政策，围绕提高自主创新能力发展产业，围绕地方优势发展产业，加强产业配套，努力实现产业集约式扩张。坚持不懈地推进改革开放。进一步深化经济和行政体制改革，更加注重运用经济手段和法律手段，充分发挥市场配置资源的基础性作用，创造性开展工作，不断破解发展中存在的各种难题。继续实施大开放主战略，拓宽领域，提升水平，为经济发展注入新的活力。坚持不懈地建设和谐社会。牢固树立以人为本，执政为民的思想，加强民主法制建设。正确处理改革、发展和稳定的关系，协调社会各方面利益关系，切实维护广大人民群众的根本利益。

三、2005年政府主要工作任务和措施

（一）进一步做好“三农”工作，保持农业和农村发展的好势头

做好农业、农村、农民工作，一要抓政策落实。全面贯彻落实中央和省委出台的今年一号文件精神，稳定、完善和强化各项支农政策，做到已经实行的政策不变，已给农民的实惠不减，支农措施的力度只能加大，不能减弱。从今年起，农业税全免，实行农民合同内“零”负担。继续实施种粮直补、良种补贴和农机具购置补贴，对稻谷实行最低收购价。今年全市粮食播种面积稳定在156.74万亩，粮食总产稳定在11.24亿斤。二要抓农业投入。抓好贵溪市优质粮食产业项目的申报和实施，力争贵溪列入全国农村能源建设项目县。认真组织实施白塔渠挖潜改造、中潢圩堤除险加固和万亩圩堤土方断面达标工程，完成35座病险水库除险加固任务，打造雷溪、潢溪两个园田化建设示范点。继续实施好林业重点生态工程，着力抓好林业产业体系建设。加快农村公路建设步伐，全年完成乡村公路新建、改建280公里。实施好画桥、上清等国家投资的土地开发整理项目。三要抓农民增收。以市场为导向，扶持做强农业产业化龙头企业，力争全市农业产业化龙头企业实现销售收入增长20%。积极推广良种良法，加强对农民的科技培训，提高农民科技致富水平。按照“山上办绿色银行，山下建优质粮仓，水面兴特色养殖”的思路，调整优化产业布局，狠抓农业精品项目和亮点工程。重点打造10万亩苗木花卉基地、白鹤湖农业生态园两个亮点，继续做强贵北果业、贵南笋竹、锦南白塔葛、锦中苗木花卉、瘦肉型猪等五大优势产业。建立一批无公害、绿色、有机农产品示范基地。扩大劳务输出规模，增加农民非农收入。积极引导小城镇健康发展，重点抓好锦江国家级小城镇和邓埠等6个省级重点镇建设。大力扶持和发展农村合作经济组织，广开农民增收渠道。四要抓农村稳定。进一步减轻农民负担，抓好防汛、抗旱、森林防火和防疫等工作。依法调处土地、山林、水利权属纠纷，维护群众的合法权益，确保农村稳定。

（二）保持工业发展的强劲势头，推进产业结构优化升级

走新型工业化路子，要坚持以优势特色产业为主导，以骨干企业为龙头，以工业园区为载体，以科技创新为动力，加快产业集聚，延长优势产业链，促进工业经济做强做大。2005年规模以上工业增长速度要力争超过20%。一要大力培育特色优势产业。携手江铜，打造“铜都”产业优势。倾力支持江铜四期技改扩建30万吨阴极铜和22万吨铜材加工等项目建设，争取规模大、科技含量高的铜加工项目相对集中放在鹰潭，做大做强以铜为主的国家新材料产业化示范基地。支持耐乐、金泉、亚菲达、江西铜材、华锋、华泰、海燕、安固等企业铜材加工项目的实施，围绕铜材、精密制造、医药、化工、眼镜等优势特色产业，搞好配套和服务，增强产业集聚效应。二要全力支持重点企业加快发展。支持重点企业依靠科技进步，提高自主创新能力。及时帮助解决企业发展中遇到的重大困难和问题，努力破解电力供应、交通运输、流动资金等制约因素，为企业发展提供良好的环境支持、政策引导和协调服务。三要加快工业园区基础设施配套建设。全面建成市工业园“四纵四横”交通网络，完成贵溪220千伏变电站和余江工业园110千伏变电站建设，不断完善水、电、通讯等配套设施。四要着力推进企业技术进步。重点抓好喜泰电机等10个重点技术改造项目和异型铜管等10项高附加值新产品的研制开发，建立项目责任制，全过程跟踪服务，增强企业发展后劲。

（三）大力发展旅游业，促进第三产业全面发展

进一步完善“以市区为主体，龙虎山景区为龙头，贵溪、余江为两翼，道教文化为特色，吃、住、行、游、娱、购相配套，景区市区联动，旅游文化联动”的大旅游格局。落实龙虎山旅游恳谈会的好思路、好建议，加快改革，整合资源，突出主题，打响品牌。深入开展创建中国优秀旅游城市活动，明确责任，加大投入，狠抓落实。举办2005年中国（鹰潭）道教文化节暨第九届龙虎山文化旅游节，举办海峡两岸道教文化论坛。积极申报龙虎山世界地质公园，做好申报世界自然、文化“双遗产”的准备工作。加大宣传促销力度，强化媒体宣传。以上海、浙江、福建、江苏等华东市场为重点，搞好公众营销和业内促销。挖掘道教文化底蕴，支持办好天师府建府900周年庆典。做好角山文化遗存申报国家重点文物保护单位工作。抓紧《龙虎山风景名胜区总体规划》修编工作。做精江铜工业游、农业观光游等新线路。加快上清古镇改造，争创全国历史文化名镇。加快天门山二期、天鹅湖一期、上清宫二期、洪五湖、香炉峰等景点建设。提升旅游配套产业，延伸旅游产业链。开发特色饮食、特色娱乐、特色商品和特色纪念品。加强旅游行业管理，提高旅游服务水平。龙虎山景区接待游客达到150万人次，旅游直接收入4800万元。

依托交通优势，加快发展现代物流和商贸服务业。培育和繁荣南站果蔬、水产品、五金等批发市场，完善市场服务功能，扩大商品辐射半径。规划建设饮食一条街、粮食加工、汽车及配件市场，创造条件开工建设鹰潭物流中心。大力发展物流配送、通信、金融、保险、会计、咨询、法律等现代服务业。加快培育新的消费热点，促进住房、教育、体育健身、文化娱乐和卫生保健消费。开拓农村消费市场，增强消费对经济增长的拉动力。加强市场监管，继续开展以流通领域食品药品安全为重点的市场整治活动，努力创造良好的消费环境。

（四）围绕创“四城”活动，推动城市建设再上新台阶

扎实开展创建省卫生城市、省园林城市、中国优秀旅游城市和全国双拥模范城市活动，调动方方面面的积极性，推动城市规划、建设和管理上台阶。坚持科学规划，提高城市品位。严格按照城市总体规划办事，分步推动城市框架拉大进程，完善城市功能配套。组织编制《鹰潭市城镇体系规划》，完成南站东区和夏埠区控制性详规修编，编制城市道路交通系统、城市绿地系统以及城市消防、防洪系统等专项规划。扎实推进胜利文化休闲广场、东湖景观二期工程和沿江大道景观工程建设，改造鹰潭人民公园、梅园月牙湖。启动320国道鹰潭市区段拓宽工程、龙虎山南大道建设和206国道鹰西至龙虎山段改造工程，建成51号路、院里路，建设42号、46号道路。加快污水处理工程和垃圾填埋场建设速度，完成3.5万吨自来水扩建工程。力争市消防指挥中心建成投入使用。启动沿江小区开发，完成院里小区二期（安居工程）等一批住宅小区建设。放宽户口准入条件，扩充城市人口，提高城市化率。加强城市管理，强化社区服务功能。积极探索城市管理综合执法，建立城市管理长效运行机制。完善和落实规划责任追究、规划听证、规划审批程序等制度。重点抓好占道经营和违章建筑的整治，打击摩的非法营运。加强社区基础设施建设，完成小街小巷的硬化和亮化。强化社区就业服务、卫生服务、医疗服务、文化娱乐服务等社会功能。

（五）实施项目带动战略，进一步提高对外开放水平

抓项目促发展。切实组织好全市“十一五”规划和重大项目的编制和论证工作。建立和完善市、县、行业主管部门和企事业单位四级项目库，受益财政拿出一定资金，扎实推进项目前期工作，力争尽可能多的项目进入国家和省“十一五”规划盘子。继续大力支持贵电二期扩建工程，确保两台机组年内投产。支持浙赣铁路电气化改造、黄金埠电厂专用铁路线、鹰景高速公路等国家和省重点工程建设。积极争取对鹰厦铁路、皖赣铁路进行改造。开工建设贵溪入闽公路。抓环境促招商。认真组织实施“重大项目招商引资年”活动，围绕工业、旅游、农业、基础设施、科教文卫“五条线”开展重点招商，千方百计引进一批科技含量高、带动性强、投资规模大的工业项目。大力开展“诚信优质服务年”活动，建立重大项目推进调度会议制度和引资项目跟踪服务责任制，完善客商投诉考核制度和客商评议评价制度，兑现各项优惠政策，进一步优化招商引资环境。严厉查处对企业乱检查、乱罚款、乱摊派和向客商索拿卡要的行为。努力实现外贸出口3150万美元、引进外资3950万美元。按照新的口径，引进内资完成24亿元，其中引进省外工业项目资金17亿元。

（六）加快改革步伐，为经济发展提供持久动力和活力

大力推进企业股份制改革，加快天施康药业、三川水表、南方照明等公司的上市步伐。纵深推进国有集体企业产权制度改革。努力破解改制企业职工医保、资产变现等遗留问题，严格执行产权交易政策，防止国有资产流失。认真落实《国务院关于深化改革严格土地管理的决定》，加强土地管理，严格土地执法，推进集约用地，提高土地综合效益。进一步深化粮食流通体制改革，大力推进国有粮食企业体制和机制创新，完善粮食管理监督检查体系。加快水利工程产权制度改革。积极开展林业产权制度改革。不断推进事业单位综合改革。鼓励多种经济成份发展医疗卫生事业，推进公共医疗卫生服务机制创新。加快资本、劳动力、技术等要素市场和社会信用体系建设。深化公共财政改革。开展“乡财县代管”试点，进一步完善部门预算制度，组建国库集中支付中心，进一步规范政府采购行为。继续深化农村税费改革和各项配套改革。推进金融体制改革，重视防范和化解金融风险，加大金融对地方经济建设的支持力度。加强与省开发银行等金融机构的合作，不断拓宽融资渠道。大力鼓励、支持和引导非公有制经济发展。加大政策扶持力度，营造平等竞争环境，提高非公有制经济科技含量和市场竞争力。

（七）切实加强社会主义精神文明建设，推进各项社会事业全面进步

加强公民道德建设。结合“建设和谐平安江西，共创富民兴赣大业”主题教育活动，继续深入开展“告别陋习、树立新风”活动，进一步提高市民素质。组织开展多种形式的群众性精神文明活动，加强法制宣传和科普教育，倡导科学、文明、健康的生活方式、诚信守法的行为规范和尊老爱幼的社会风尚。推进民主法制建设。深入开展法律进农村、进社区、进学校活动，培养一批“普法型社区”、“学法型家庭”。做好“四五”普法验收工作。加强基层民主政治建设，大力推进村务公开和民主管理工作。积极推进农村计划生育利益保障机制和计划生育村民自治工作，综合整治出生人口性别比例失调问题。加强矿产、森林、水土等资源管理，杜绝非法开采、乱砍滥伐、乱采滥挖。控制环境污染，严格执行环保设施与项目建设“三同时”的要求，确保完成“十五”环保各项指标任务。

深入推进人才强市、科教兴市战略。树立人才是第一资源的观念，完善人才培养、吸引和使用机制，围绕我市经济和社会发展的需要，引进各类高层次人才。着力抓好全国科技进步示范市、国家新材料产业化示范基地和全省制造业信息化示范市建设“三大工程”。进一步巩固提高基础教育，在全市农村中小学普遍推行“一费制”，将农村义务教育公用经费补助标准提高一倍。加强农村学校危房改造力度，消灭农村中小学现存一类危房。努力将农村中小学辍学率分别控制在1.5%和0.3%以下。大力推进优质高中建设，积极发展学前教育、职业教育和高等教育，支持和规范民办教育。积极推进文化产业化进程，继续实施小康文化工程、文化精品工程和百县千乡文化工程。推进“五个一”精品工程创作，加强文化和新闻出版市场管理。建立健全医疗保健服务体系、疾病预防控制体系、卫生执法监督体系和突发公共卫生事件医疗救治体系。加快市人民医院门诊医技楼、市急救中心和市、县传染病治疗机构建设。推进乡镇卫生院基础设施建设和管理体制改革。引导和规范社会力量办医。加强食品、药品和医疗市场监管，保障人民群众食品、药品和医疗安全。进一步加强体育工作。加快市体育中心建设，积极备战省第十二届运动会，以《全民健身计划纲要》颁布10周年为契机，进一步推动全民健身活动的开展。加大农村有线电视“村村通”工程建设力度，力争全市80%的乡镇、30%的行政村开通有线电视。广泛开展国防教育，做好民兵预备役和人防工作，完成年度征兵任务。加强与部队、铁路等驻市单位的沟通合作，促进驻市单位与地方建设互惠互利，共同发展。

（八）关心群众生活，努力构建和谐平安鹰潭

千方百计扩大就业再就业。把充分就业作为经济社会发展的优先目标和政府工作的重要职责，实行更加积极的就业政策。建立促进就业再就业工作长效机制。加强就业培训，扩大劳务输出，引导民工有序流动，充分就业。年内新增就业岗位1.8万个，输出劳力16万人。切实做好高校毕业生就业和退伍转业军人安置工作。进一步推进社会保障体系建设。继续巩固“两个确保”，做好“三条保障线”的衔接。积极推进国有企业下岗职工基本生活保障与失业保险并轨。进一步做好企业离退休人员养老金按时足额发放和城市低保对象应保尽保工作，确保离退休人员和困难群众的基本生活。抓紧出台鹰潭市城镇职工基本医疗保险实施办法，研究完善灵活就业人员参保办法。积极开展清理拖欠农民工工资、规范劳动力市场秩序和劳动保护执法监察等工作，维护劳资双方合法权益。进一步完善城乡一体的社会救助体系，健全特困群众生活救助制度。提高农村五保集中供养率。关注残疾人事业和生活。大力开展结对帮困和社会捐助工作。高度重视和切实加强信访工作，积极预防和妥善处置群体性事件。认真处理好农村征地、城镇房屋拆迁、国有企业改制、案件审理等涉及群众切身利益的问题，把矛盾解决在基层。强化基层综合治理网络建设，进一步完善治安防控体系。严厉打击刑事犯罪，坚决遏制刑事案件多发的势头。坚持不懈地抓好生产安全、交通安全、防火安全、学校安全、旅游安全等安全工作，确保国家和人民群众生命财产安全。

四、加强政府自身建设，提高政府行政能力

组织完成好今年繁重而艰巨的任务，关键是要提高行政能力，努力建设法治政府、责任政府、诚信政府、服务型政府和廉洁政府。

坚持科学理政，进一步转变政府职能。认真落实科学发展观和正确政绩观，努力遵循经济规律和执政规律办事，加快政府由管理型向服务型转变，进一步把政府职能转到经济调节、市场监管、社会管理和公共服务上来，精心营造良好发展环境，不断提高政府效能和为民服务水平。

坚持民主施政，促进政府工作民主化进程。完善公众参与、专家论证和政府决策相结合的科学民主决策机制，建立健全重大决策立项调研、集体决策、专家咨询、社会公示和听证、决策失误责任追究等制度。全面推行政务公开，建设“阳光政府”，保障人民群众的知情权、参与权和监督权。依法受理群众投诉，诚恳接受群众评议，制定并实施《影响机关效能行为责任追究办法》，规范公务员行政行为。

坚持依法行政，把政府各项工作纳入法治轨道。认真贯彻实施《行政许可法》和国务院《全面推进依法行政实施纲要》，进一步深化审批制度改革，简化审批手续。精简完善政府工作程序，提高政府公文的质量和实效性。严格按照法定权限和程序行使职权，进一步加强和改革行政执法，强化行政执法监督，保护人民群众的合法权益。

坚持从严治政，建设为民、务实、清廉的公务员队伍。切实加强政风建设，大兴求真务实之风，坚决反对形式主义、官僚主义，重实干、办实事，求实效。认真开展保持共产党员先进性教育活动。加强学习、培训和多岗位锻炼，全面提升公务员队伍整体素质,增强责任感和事业心。牢记“两个务必”， 树立艰苦奋斗、干净干事的事业观。落实党风廉政责任制，自觉接受监督，严肃查处各类违法违纪行为，树立政府清政廉洁的良好形象。

各位代表，时代赋予我们重任，人民寄予我们厚望。我们要倍加珍惜历史机遇，倍加珍惜鹰潭心齐、气顺、劲足的大好局面，在省委、省政府和市委的正确领导下，同心同德、开拓前进，共同创造鹰潭更加美好的明天！
